# Regional differences in rapid evolution during severe drought

**DOI:** 10.1002/evl3.218

**Published:** 2021-02-23

**Authors:** Daniel N. Anstett, Haley A. Branch, Amy L. Angert

**Affiliations:** ^1^ Biodiversity Research Centre and Department of Botany University of British Columbia Vancouver British Columbia V6T 1Z4 Canada; ^2^ Department of Zoology University of British Columbia Vancouver British Columbia V6T 1Z4 Canada

**Keywords:** Adaptation, climate change, dehydration avoidance, dehydration escape, *Erythranthe cardinalis*, flowering time, plasticity, resurrection study, specific leaf area

## Abstract

Climate change is increasing drought intensity, threatening biodiversity. Rapid evolution of drought adaptations might be required for population persistence, particularly in rear‐edge populations that may already be closer to physiological limits. Resurrection studies are a useful tool to assess adaptation to climate change, yet these studies rarely encompass the geographic range of a species. Here, we sampled 11 populations of scarlet monkeyflower (*Mimulus cardinalis*), collecting seeds across the plants’ northern, central, and southern range to track trait evolution from the lowest to the greatest moisture anomaly over a 7‐year period. We grew families generated from these populations across well‐watered and terminal drought treatments in a greenhouse and quantified five traits associated with dehydration escape and avoidance. When considering pre‐drought to peak‐drought phenotypes, we find that later date of flowering evolved across the range of *M. cardinalis*, suggesting a shift away from dehydration escape. Instead, traits consistent with dehydration avoidance evolved, with smaller and/or thicker leaves evolving in central and southern regions. The southern region also saw a loss of plasticity in these leaf traits by the peak of the drought, whereas flowering time remained plastic across all regions. This observed shift in traits from escape to avoidance occurred only in certain regions, revealing the importance of geographic context when examining adaptations to climate change.

**Impact Summary**

Evolutionary biologists have known for decades that evolution can occur quickly, over just a few generations. This generates hope that species will be able to evolve in response to climate change and avoid local extinction. Populations at species’ range edges are critical because they may represent populations that are both the most vulnerable and most likely to contribute genetic material to climate‐driven range expansions. Yet systematic assessments of rapid evolution across the range of a species remain rare, especially when using a resurrection study. Resurrection studies are experiments where propagules sampled from the same localities across different time periods are grown in a common environment, allowing for a rigorous measurement of evolutionary change. This study is unique in scope because we carry out range‐wide comparisons and maintain a mostly intact time series, measuring adaptations at a regional scale before the start of the drought and every subsequent year until the drought's peak. This allows us to chronicle the evolutionary impacts of the strongest sustained drought in the western USA in thousands of years. Our results suggest that populations at the southern end of the range of scarlet monkeyflower shifted drought adaptation strategies, from phenological traits that foster escaping drought toward morphological traits that promote dehydration avoidance. Southern populations also became less plastic in leaf morphological traits, whereas northern regions showed little change in leaf morphology. Overall, our work shows how vulnerable populations at southern range edges evolved rapidly, while northern regions did not. This lack of evolution may be due to less evolutionary pressure or lack of sufficient genetic variation.

Climate change is a global biodiversity threat with the potential to disrupt the health, persistence, and distribution of natural populations and communities (Parmesan and Yohe 2003; IPCC 2014; Pecl et al. 2017). The rapid rate of climate change can make populations vulnerable, leading to possible extirpations and shifts in species’ distributions (Chen et al. 2011; Dullinger et al. 2012; Dai 2013; Diffenbaugh & Field 2013; Panetta et al. 2018). Within environments that naturally experience pronounced cyclical changes across years, climate change is set to exaggerate these impacts (Trenberth *et al*. 2014), introducing record‐setting extremes and decreasing predictability. For example, anthropogenic warming turned what would have been a moderate drought in the Western USA into the most severe drought in over 400 years (Williams et al. 2020). In California, 2012–2015 was the most drought‐impacted four‐year period in the history of record keeping and had no close analog in thousands of years of tree ring records (Robeson 2015). These extreme events are likely to increase mortality and threaten population stability (Allen et al. 2010; Moran et al. 2014), particularly for populations at the extreme ends of a species’ range.

Sudden changes or increases in environmental variability could result in maladaptation to present environments. To reverse population decline that could lead to local extinction, rapid evolution might be required to restore net reproductive rates above replacement (Gomulkiewicz & Holt 1995; Bell 2013). In recent decades the prevalence of rapid evolutionary responses has become apparent (Reznick et al. 1990; Hairston Jr et al. 2005; Agrawal et al. 2012; Grant & Grant 2014), suggesting that rapid evolution could allow for evolutionary rescue. One powerful approach to detect rapid evolution is a resurrection study, where ancestral propagules are stored and later compared to descendants in a common environment (Davison & Reiling 1995; Hairston et al. 1999; Sultan et al. 2013; Franks et al. 2018). This approach has been successful in documenting rapid evolution. For example, annual plant adaptations to drought have been found to evolve within seven years or less (Franks et al. 2007; Franks & Weis 2008; Franks et al. 2016; Dickman et al. 2019). These findings suggest that rapid evolution to climate change can occur and might be a common response in short‐lived organisms.

In a climate change context, we know less about how phenotypic plasticity may complement rapid evolution or itself evolve as a trait. Yet the rapid evolution of traits on their own may be insufficient to keep up with changes occurring from one season to the next or within a season. Phenotypic plasticity might promote population persistence by directing development towards a more drought‐adapted trait space (Nicotra et al. 2010; Richardson et al. 2017), and could even lead to the evolution of increased plasticity (Sultan et al. 2013). Alternatively, environmental extremes could select so strongly for stress adaptations that populations evolve decreased plasticity, with traits better adapted to an extreme environmental disturbance, but less plastic to future variability (Matesanz et al. 2010a). Resurrection studies crossed with an experimental treatment (e.g., dry vs. well‐watered) present an opportunity to assess changes in trait means and their plasticity (Franks 2011).

Environmental, historical, and demographic legacies likely influence if and how strongly populations adapt to climate change, and what traits evolve (Levins 1968; Parmesan 2006). Across the range of a species, populations face different environmental pressures and varying impacts of climate change (Hampe & Petit 2005; Chen et al. 2011; Dickman et al. 2019). Under these different conditions, varying dispersal, population size, and historical population bottlenecks might advance or limit evolution by natural selection (Antonovics 1976). These population attributes could vary systematically by region, depending on their location within the geographic range (Sexton et al. 2009). Thus understanding the full impact of climate change on a species requires sampling populations from across the range and investigating how adaptations to past climates impact present‐day adaptability (Leimu & Fischer 2008).

Populations at range edges might vary in the amount of genetic variation and isolation and thus their propensity for adaptive evolution (Goldberg & Lande 2007; Eckert et al. 2008). Leading‐edge populations, where range expansion has recently occurred, might have reduced genetic variation and harbor “expansion load” due to serial founder events (Peischl et al. 2013; Peischl et al. 2015). Meanwhile, rear‐edge populations are often already close to their physiological abiotic limits, and climate change could cause shifts in trait optima beyond these limits, leading to declines and extirpations without plasticity or evolutionary adaptations (Jump et al. 2006; Jump et al. 2010). If these range‐edge populations are relictual, they may have large population sizes and harbor larger amounts of genetic diversity (Hampe & Petit 2005) and thus have enough genetic variation and demographic stability to undergo rapid evolution and evolutionary rescue. Alternatively, these range‐edge populations may be trailing, suffering declining population sizes and low genetic variation, and thus at greater risk of extirpation (Hampe & Jump 2011). Comparing leading‐edge and rear‐edge populations to the range center will aid in determining how effectively a species is adapting to climate change. Rapid adaptation has been studied across two sites that varied in water availability, but were geographically proximate (Franks et al. 2007; Hamann et al. 2018); and across a species with a limited range (Dickman et al. 2019). However, the lack of resurrection experiments investigating how adaptation to drought varies across species with a broad latitudinal range remains a significant gap.

Under increasingly intense droughts, a variety of adaptations to mitigate the negative fitness consequences of drought could be favored. Suites of traits that mitigate drought are typically grouped into three “strategies”: phenological escape, morphological or behavioral avoidance, and biochemical tolerance (e.g., osmotic adjustment, stabilization with heat shock proteins) (Kooyers 2015; Volaire 2018). In plants, dehydration escape is characterized by earlier phenology, with plants developing and growing quickly enough to flower and produce seed before a terminal drought. The high genetic variation often found in phenological traits might allow for escape to readily evolve (Rathcke & Lacey 1985; Ollerton & Lack 1992; Quinn & Wetherington 2002), particularly when drought onset occurs relatively later in the season (Franks et al. 2007). However, if drought onset is too early and the plant is already close to its physiological limit, dehydration avoidance could leverage traits that decrease water loss, increasing water‐use efficiency (Volaire 2018). Dehydration avoidance allows for a longer time during which the plant can produce seeds, potentially allowing for greater seed output, and can be beneficial when drought is too sustained to escape. Ideally, a population would be able to evolve both dehydration escape and avoidance, but achieving a rapid growth rate often comes at a cost of lower water‐use efficiency, making it difficult to evolve both strategies (Geber & Dawson 1990; Ackerly et al. 2000; McKay et al. 2003; but see Kooyers et al. 2015).

Here we use *Mimulus (Erythranthe) cardinalis* (Phrymaceae) to examine variation in rapid evolution to extreme drought across a latitudinal gradient. With a resurrection approach, we assess the trajectory of drought adaptations using range‐wide seed collections carried out throughout the recent severe drought in California and Oregon. For eleven populations sampled across 7 years and grown in a greenhouse drought experiment, we measure changes in means and plasticity of five traits related to dehydration escape and avoidance (date of first flower, specific leaf area, leaf water content, carbon assimilation, and stomatal conductance) to ask: (1) Is *M. cardinalis* able to rapidly evolve in response to severe drought? (2) Do traits consistent with escape or avoidance strategies evolve in response to the drought? (3) How does rapid adaptation to drought vary across the range of the plant? We hypothesize a shift in the evolution of drought‐response strategies, with the shorter season length pushing southern and central populations towards their phenological limits, thus requiring an increase in dehydration avoidance (Hamann et al. 2018). In contrast, northern populations in southern Oregon and northern California experienced severe drought but climatic conditions on the whole were further away from physiological and demographic limits (Muir & Angert 2017; Sheth & Angert 2018; Williams et al. 2020). Thus, we hypothesize the northern populations will evolve traits that promote dehydration escape. Given the variability of water availability across the range of *M. cardinalis* both within a growing season and across the lifetime of this perennial species, we also expect to find plasticity in escape and avoidance traits across the range of the plant, but especially in southern California given high historical levels of precipitation variability in this region (Fig. S1). Through these range‐wide explorations of adaptation to drought, this study provides insight into geographic variability in the magnitude and direction of evolutionary change in response to climate change, with significant implications for the survival of populations at range edges.

## Methods

### STUDY SYSTEM

*Mimulus cardinalis* is a rhizomatous perennial herb native to lowland and montane riparian habitats (0‐2400 m) throughout California and Southern Oregon, USA and Northern Baja California, Mexico (Jepson 1993). The species is hummingbird pollinated, yet self‐compatible with high potential for geitonogamy. It propagates vegetatively through rhizomes and sexually through seeds. The forb grows in variably sized populations in immediate proximity to seeps and streambanks. Its growing season depends on water availability from rain and snowmelt and often ends with a terminal drought. The range of *M. cardinalis* lies on an aridity gradient, with southern populations experiencing historically higher temperatures, lower annual precipitation, and greater precipitation variability compared to Northern California and Oregon (Fig. S1). This gradient may have driven historical local adaptation in *M. cardinalis*, with greater growth and photosynthesis rates (Muir & Angert 2017) and faster life history (Sheth & Angert 2018) in southern populations.

### POPULATION SAMPLING

**Figure 1:**
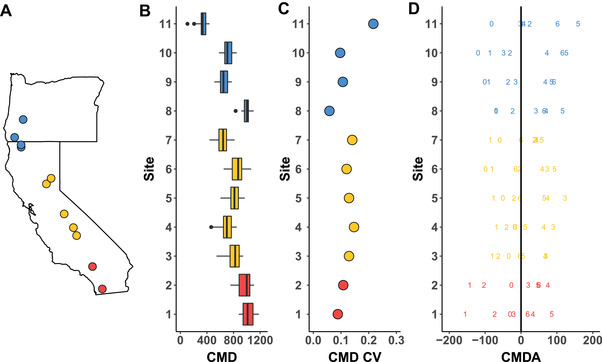
Climatic moisture deficit (CMD) across the range of *M. cardinalis* during historical conditions and extreme drought. Sites are arranged by latitude and color coded by region (blue = North, orange = Center, red = South). (A) 11 populations of *M. cardinalis* included in this resurrection study spanning California and Southern Oregon. (B) The median and distribution of yearly CMD for each year between 1979 and 2009 for each site. Boxes represent the interquartile range, while the black line is the median. Dots are years further than 1.5 times the interquartile range. (C) Coefficient of variation for CMD experienced between 1979 and 2009. (D) CMD anomaly (CMDA) during the studied drought cycle. The single‐digit numbers represent the last digit of the year (e.g., 0 = 2010, 4 = 2014). The black line delineates the historical average (1979‐2009) for each site. Higher CMD implies a drier site, while positive CMDA implies the site is drier than the historical average.

Seeds were collected from 12 populations (but see below) spanning 11° latitude from Southern California to Southern Oregon from 2010 to 2016, representing a period of record‐setting drought across the northern, central, and southern range of *M. cardinalis* (Fig. 1; Fig. S1; Table S1). Within each population, sampling occurred at the furthest downstream location, which allows for the most complete samples of the local gene pool because of the presumed prevalence of downstream dispersal. Mature, indehiscent fruits were collected, dried, and stored at 21°C. We sampled up to 10 maternal lines from most year/site combinations. We were occasionally prevented from sampling some site–year combinations due to forest fires, floods, and/or population decline.

We carried out a refresher generation (Franks et al. 2018) under greenhouse conditions (27°C day, 15°C night, 12‐h days) to minimize maternal and seed age effects. Germination rate per fruit sampled varied substantially across sites and years (Table S2, Fig. S2), but – critically – did not follow a declining trend for older samples that would be characteristic of the invisible fraction problem (Weis 2018; Franks et al. 2019). Instead, some site‐year combinations had low viability because sampling was initially conducted to quantify seeds per fruit to parameterize demographic models, and were only later used for resurrection, thus sampling emphasized indehiscent but sometimes immature fruits. The successfully germinated accessions were crossed within each site‐by‐year combination, ensuring each accession was dam and sire of one cross. This led to seeds from 385 unique families representing the sampled gene pool from each site and year.

### PLANT PROPAGATION

Eight replicates of each unique family were grown in a greenhouse at the University of British Columbia campus in Vancouver, Canada (N = 3080 plants). Multiple seeds from each family were sown over a 3‐day period on 0.25 L square pots filled with sand (Quikrete Play Sand, Georgia, USA), mimicking a riparian substrate. Cotton rounds in the bottom of each pot prevented sand loss during watering. Replicates were randomized into eight blocks across four watering tables. Bottom watering four times daily and misting were initiated at the same time for all replicates (day 0 of the experiment). Each family germinated over 2–3 weeks. Misting was halted after six weeks, at which point five timed‐release fertilizer pellets (Nutricote, T100, 14‐13‐13) were placed on the surface of each pot. Seedlings in excess of one per pot were thinned 8 weeks after sowing. Bamboo stakes were then placed in each pot to provide support for each plant.

### DRY TREAMENT EXPERIMENT

An experimental dry‐down treatment was initiated 81 days after the start of the experiment. Four blocks were kept on the same watering schedule as above and four were assigned a dry treatment, alternating tables to stratify across microvariation in greenhouse lighting and temperature. In the dry treatment, watering was cut from four to two times daily on day 82, to once daily on day 96, and to no watering after day 111 to emulate decreased waterflow and availability during stream recession that creates a terminal drought in nature. The experiment was ended on day 126.

### TRAITS ASSOCIATED WITH ESCAPE AND AVOIDANCE

We assessed a series of traits that are known adaptations for dehydration escape and avoidance. Earlier flowering time contributes to dehydration escape while later flowering time can be involved in drought avoidance through increased water use efficiency associated with slower growth. We assessed the date of flowering daily throughout the entire growth period. We also sampled one leaf per plant for all leaf trait measurements to ensure significant leaf removal did not impact further trait collection. Leaves were collected in three out of the four replicates across both well‐watered and drought treatments. Leaves were consistently sampled at the sixth to eighth node (counting from the bottom) depending on leaf size in order to ensure the size was sufficient for photosynthetic assessments. Specific leaf area (SLA) and water content were also assessed, where lower SLA and greater water content were assumed to indicate dehydration avoidance, while greater SLA would contribute to dehydration escape (Fonseca et al. 2000; Kooyers et al. 2015). Both traits were assessed 15 to 22 days after the initiation of the dry treatment by measuring leaf area, and wet and dry mass. SLA was calculated as wet leaf area/dry leaf mass (cm^2^/g) and water content was calculated as wet leaf mass/dry leaf mass (%).

We further assessed drought adaptations by measuring the carbon assimilation rate and stomatal conductance. High values of carbon assimilation and stomatal conductance indicate dehydration escape, because plants growing quickly require higher levels of carbon input and an increased supply of carbon dioxide. Lower values indicate dehydration avoidance, because stomata are closed more frequently to prevent water loss, which can decrease carbon assimilation and growth rate. Using a LI‐6800 portable photosynthesis system (LICOR INC, Lincoln, NE, USA) we conducted point measurements on the most recent fully expanded leaf (at the 6th to 8th node). The leaf cuvette was set to 400 ppm CO_2_, 1200 μmol m^−2^ s^−1^ light intensity, and approximately 50% relative humidity. A single leaf was placed into the cuvette and three measurements were recorded over approximately 1 min once stability criteria were met. These three measurements were averaged for subsequent data analysis. Two of the four replicates per treatment level were measured, due to time limitations of using the LI‐6800. Plants that were too small to fit into the chamber were excluded.

### CLIMATIC DATA AND SITE/YEAR SELECTION

Monthly climate data were downloaded from Climate WNA (Wang et al. 2016) for each of the 12 sites from 1979 to 2016. We focused on mean annual temperature (MAT), mean annual precipitation (MAP), and Hargreaves climatic moisture deficit (CMD). Because *M. cardinalis* senesces by the end of September, we calculated yearly values of environmental variables by averaging (MAT) or summing (CMD, MAP) for each monthly value for January to September of the focal year and October to December of the previous year. We calculated historical climate averages for September 1979 to October 2009 as well as anomalies for each sampled year (climate variable for given sampling year – historical average) (Fig. 1; Fig. S1). We also calculated the coefficient of variation across the 30‐year historical data for each site by taking the fraction of variance/mean for each climatic variable. Due to the nature of a Mediterranean climate this 30‐year period included wet and dry cycles and thus provided a reasonable baseline for the conditions experienced by our study system before our study period. To further illustrate the impact of drought we also present Palmer Drought Severity Index downloaded from NOAA (Vose et al. 2014) for five drainage divisions that encompass our study sites (California: South Coast, San Joaquin, Sacramento, North Coast; and Southwest Oregon).

Because PDSI was not available at the site level, we focus on site‐level CMD ‐ the sum of differences between monthly precipitation and atmospheric evaporative demand ‐ to capture the additional soil moisture required to avoid drought (Wang et al. 2012). For each site, we used the CMD anomaly (CMDA) to focus analyses on the linear portion of the time series that would best test adaptation to drought, from the lowest to highest drought‐impacted year, without reversals. This was important because sites differed in the exact onset, duration, and cessation of drought, making calendar years a poor proxy for the extent of drought‐induced selection. However, here we analyze patterns over time, rather than using climatic parameters like CMDA as explanatory variables, because time captures the cumulative impacts of successive years of drought‐induced selection. By considering anomalies in CMD, we were able to most effectively quantify the impact of the extreme drought event within each site relative to its 30‐year average. Based on this information, we selected a subset of site‐year combinations that began with the lowest CMDA and ended with the highest CMDA (Fig. S3). For most sites this meant ending the data set in 2014 or 2015 rather than 2016, since the peak of the drought occurred in these earlier years. As well, 2011 had lower CMDA than 2010 for some sites, and thus 2010 was sometimes excluded. We excluded one site entirely since we were missing seeds both from the lowest and highest CMDA levels. Therefore, the final data set included 2144 plants from 268 families across 11 sites. We then grouped these sites into three distinct regions capturing the northern, central, and southern range of *M. cardinalis* (Fig. 1; Table S1).

### STATISTICAL ANALYSIS

All plant traits were assessed for normality and did not require transformations. To test for regional (north, centre, south) and treatment differences (wet, dry) across time (year of seed collection) we carried out mixed models in R (R Development Core Team 2020) using the *lmer* command in the lme4 package (Bates *et al*. 2015). For each trait, we began with a full model of Region*Treatment*Year with family, block, and site as random intercepts. Significance was assessed by comparing this 3‐way model to a model with all 2‐way interactions using *lrtest* in the lmtest package (Hothorn et al. 2019), where significantly higher likelihood values led to the selection of the supported model. When there was no significant difference between models, the simpler model was selected. We retained models with marginally significant results (0.05< p< 0.07) but indicate weaker confidence in them. For each trait, we compared all possible simpler models until we arrived at a model that was significantly favored over the next reduced model (Table S3). To visualize the patterns we used *visreg* (Breheny et al. 2020) extracting the residuals for Region*Year*Treatment models, and plotting linear models for wet and dry treatments across years for every region using *ggplot2* (Wickham 2011). For each trait and region, we also plotted model‐estimated slopes (and their standard errors) for trait change over time as expressed within the dry and wet treatments using values from *visreg* in *ggplot2*. To test for possible differences due to number of years sampled between regions, we thinned the data set to only the first selected pre‐drought year and the peak drought year for each population. We re‐ran all aforementioned analyses and found no important statistical or visual differences between results and hence only present the full data set.

## Results

### CLIMATE

A severe drought occurred between 2012 and 2016, with peak precipitation anomalies in 2014 (Fig.S1C), and peak temperature anomalies in 2015 (Fig. S1F). Yet this prolonged drought was not felt uniformly across California and Southern Oregon. The worst drought in recorded history occurred in Southern (Fig. S4A) and parts of Central California (Fig. S4B) with extremely low PDSI levels approaching or surpassing −8. North Central, Northern California, and Southwestern Oregon also recorded severe drought, but this drought still had modern analogs (Fig. S4C‐E). Across our 11 target sites, CMDA captured that the peak of the drought often varied across years with some sites having a clear peak, while others having multiple drought years of similar severity (e.g. Site 3, Site 7). Yet there were generalizable regional patterns with clear distinctions in MAT and MAP means and variability (Fig. S1). Indeed, central and southern sites show more temperature variability than northern ones, while a latitudinal gradient exists in precipitation variability, with increased variability towards southern sites (Fig. S1E). Overall, the impacts of changing climate are captured by MAT where 2014 and 2015 appeared as the hottest years in record keeping (Fig. S5). CMD and MAP provide our best ability to quantify the impacts of drought at the site level, although unlike the regional PDSI metric, these variables do have modern analogs in most cases (Fig. S6, S7).

### SPECIFIC LEAF AREA

**Figure 2:**
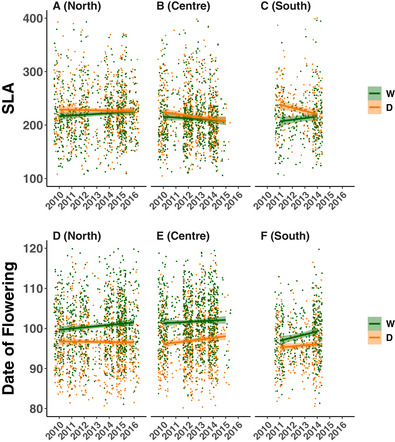
Evolution of specific leaf area (SLA) and date of flowering across the range of *M. cardinalis* from the least to most drought‐impacted year. SLA shows (A) slight increases in the North, (B) decreases in the Centre, and (C) loss of plasticity in the South. Date of flowering shows evolution of later flowering time across the (D) North, (E) Center, and (F) South. Each point represents residuals from a mixed model including Region*Year*Treatment model and Site, Family, and Block as random effects. The lines are linear models run on the residuals with 95% confidence intervals given for both wet and dry treatments.

**Figure 3:**
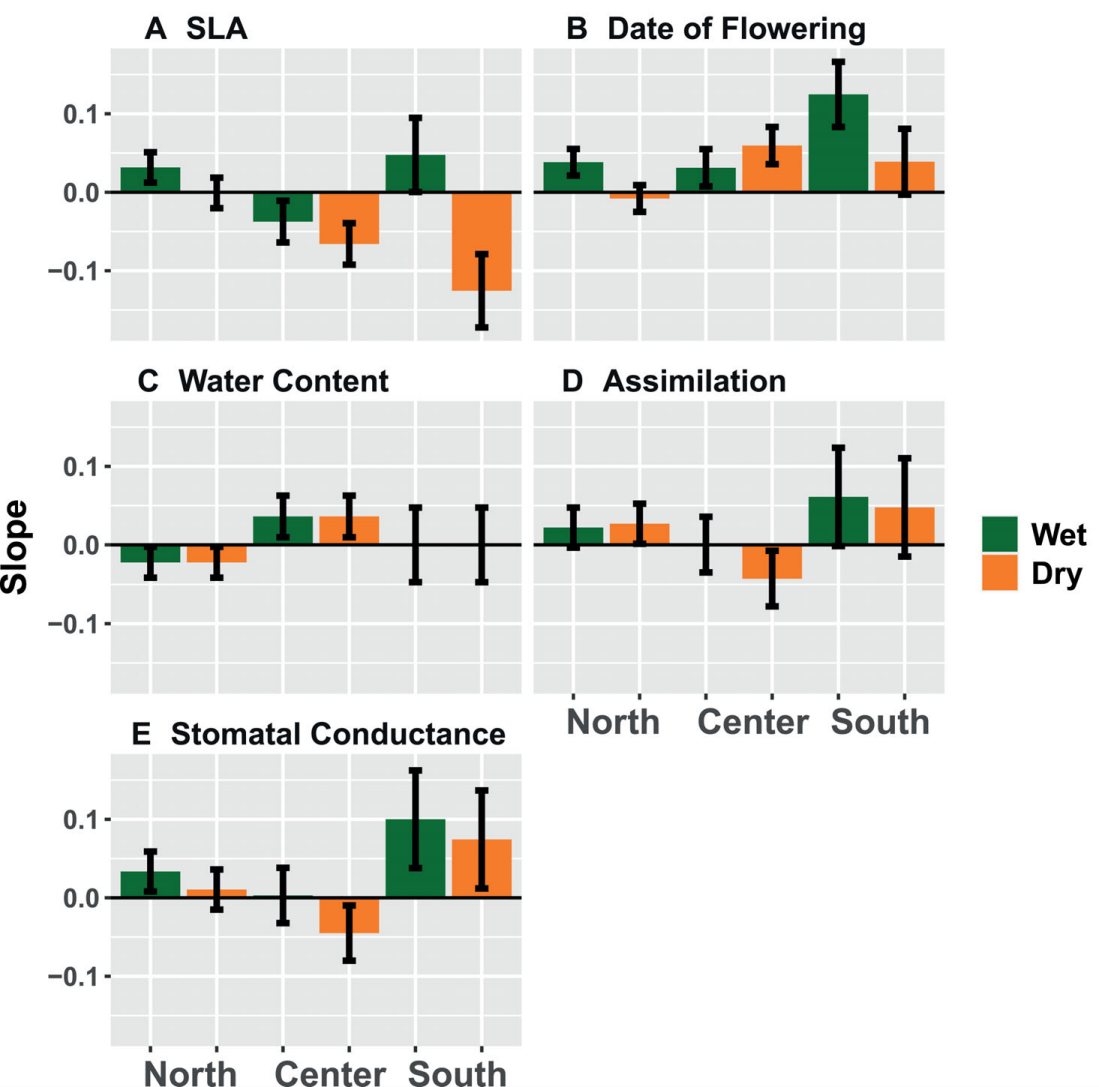
Slopes capturing the change of drought‐associated traits over time from the least to most drought‐impacted year. (A) SLA slopes vary considerably across regions and treatments and show a significant three‐way interaction (*P* = 0.003). Positive slopes represent the evolution of dehydration escape, while negative slopes are consistent with evolution toward dehydration avoidance. (B) Flowering time slopes are positive for most regions and water treatments, although slopes are not significantly different since the favored model is Region*Treatment + Year (*P* = 0.014). Positive slopes represent evolution of dehydration avoidance, while negative slopes show evolution of dehydration escape. Slopes vary much less for (C) water content (Treatment‐only model; *P* = 0.001), (D) carbon assimilation (Region*Treatment + Year; *P* = 0.06), and (E) stomatal conductance (Treatment‐only model; *P* = 0.055). Error bars show standard errors.

SLA showed geographical variation in the strength and direction of evolution through the severe drought with a significant Region*Year*Treatment interaction (*P* = 0.003; Table S3). Populations from the northern portion of the *M. cardinalis* range showed a minor increase in SLA over time in the wet treatment and no temporal trend in the dry treatment (Fig. 2A and 3A). Populations in the center of the range evolved lower SLA as the drought progressed across years in both the wet and dry treatments (Fig. 2B and 3A). In contrast, southern populations showed the evolutionary loss of plasticity, with a reduction of SLA over time in the dry treatment and an increase over time in the wet treatment (Fig. 2C and 3A).

### DATE OF FLOWERING

*Mimulus cardinalis* evolved later date of flowering in most regions and treatments. The model including Year and the Region*Treatment interaction was significant (*P* = 0.014; Table S3). Date of flowering evolved to be later over time (Fig. 2D and E). Throughout the range, there was flowering time plasticity between the wet and dry treatments, with central and northern regions having greater flowering time plasticity compared to southern regions (Region*Treatment interaction; Fig. 2D and E). In contrast to patterns for SLA, plasticity in flowering time remained throughout the drought (Fig. 2F). Furthermore, date of flowering was overall earlier in the southern part of the range.

### PHYSIOLOGICAL TRAITS

Evolutionary trajectories for water content, carbon assimilation, and stomatal conductance were not as pronounced. Water content was greater in the well‐watered treatment, with statistical support for a Treatment‐only model (*P* = 0.001; Fig. S8A; Table S3). There was a trend for greater water content in the center of the range (Fig. S8A), while evolution in water content over time was difficult to distinguish from zero (Fig. 3C). Carbon assimilation showed a marginally significant Region*Treatment + Year model (*P* = 0.06). There was a relatively weak increase in assimilation over time, and in the South, there was marginally greater assimilation in the dry treatment over the wet treatment (Fig. S8D‐F; Fig. 3D). Stomatal conductance showed only a marginally significant treatment model (*P* = 0.055; Table S3). Specifically, there was marginally greater stomatal conductance in the wet treatment (Fig. S8G‐I) and a trend for the evolution of greater stomatal conductance in the south (Fig. 3E).

## Discussion

Through a resurrection study, we found range‐wide variation in evolutionary responses to extreme climatic perturbation in *M. cardinalis*, showing that the direction and magnitude of rapid evolution to drought varies across different environmental and historical contexts. Dehydration avoidance evolved in the center and south of the range but did not show evolution in the north (Fig. 2A‐C) matching the historical drought severity observed in central and southern regions (Fig. S4). This supports the hypothesis that southern, more historically arid regions will evolve avoidance when faced with extreme drought. However, we do not find evidence of the evolution of escape in northern populations, which were historically exposed to greater precipitation. Instead, plasticity may have been sufficient in these environments. As predicted, plasticity in escape remained high across the range of *M. cardinalis*, but contrary to expectations avoidance traits like SLA showed little plasticity (Fig. 2A and B) or underwent an evolutionary loss of plasticity in southern regions (Fig. 2C). The evolution of avoidance in central and southern regions could depend both on drought severity as well as the genetic legacy of past environmental differences and local adaptation to those differences. Indeed, regions with a history of greater impacts of drought could be more likely to rapidly evolve adaptations, better positioning these populations to undergo evolutionary rescue.

### DEHYDRATION ESCAPE VERSUS AVOIDANCE

Escaping dehydration through early phenology is a common strategy to cope with drought in Mediterranean environments (Franks et al. 2007; Berger & Ludwig 2014; Hamann et al. 2018). Prior to the severe drought, we found date of flowering was earlier and SLA was higher in southern and central regions (Fig. 2A‐C), yet rather than evolving further toward traits that promote escape, the traits moved toward values consistent with dehydration avoidance. If drought had not occurred in 2012‐2016, then evolution of further drought escape might not be expected. However, the central and southern regions saw severity greater than any since recordkeeping began (Fig. S4; Robeson 2015). It is possible that earlier flowering time and greater SLA evolved in response to earlier droughts in the early to mid‐2000s since escape‐associated traits may have been sufficient during moderate drought and then simply remained in 2010 despite a few wet years. That we observed evidence for the evolution of avoidance in *M. cardinalis* suggests that escape proved to be insufficient for this perennial plant. Indeed, regions with the greatest climatic moisture anomalies (Fig. 1D) and high PDSI (Fig. S4) evolved lower SLA and later flowering time, both consistent with dehydration avoidance.

There is limited evidence of evolution of avoidance‐related trait values in resurrection experiments. Evolution of later flowering time was also found in response to drought in *Mimulus laciniatus*, although rapid evolution of germination time and SLA still supported a dehydration escape strategy (Dickman et al. 2019). There also appeared to be limits to the evolution of earlier flowering time and evolution of increased water use efficiency in the wetter of the two populations examined by Hamann et al. (2018). Yet it is *M. cardinalis* that has shown the evolution of dehydration avoidance more convincingly. *Mimulus cardinalis* is often perennial, which might make dehydration avoidance more favorable across multiple seasons. Indeed, dehydration avoidance and tolerance have been observed in longer‐lived organisms in Mediterranean climates such as oak trees in southern California (Knops & Koenig 1994). Given the widespread impacts of the severe drought, additional resurrection studies across more systems would aid in understanding if the shift from escape to avoidance is becoming more common in forbs, and if this shift will hold or return to escape after peak drought years.

Despite the evolution of lower SLA (consistent with increased drought avoidance), we did not see evidence of lower rates of leaf‐level gas exchange (photosynthetic carbon assimilation and stomatal conductance), which would be consistent with dehydration avoidance. It is possible that changes in other features of leaf morphology such as stomatal density, mesophyll and cuticle thickness, or trichomes were able to reduce water loss without impacting the frequency and duration of stomatal opening. It is also possible that populations have evolved biochemical tolerance mechanisms, allowing for maintained carbon assimilation and stomatal conductance. Leaf‐level gas exchange measurements are both time consuming and notoriously plastic (Caruso et al. 2020) leading to low replication and greater measurement error. Both of these issues can result in insufficient power to resolve real temporal trends in stomatal conductance patterns and carbon assimilation (e.g., Fig. 3F and I). However, we did find a marginally significant increase in carbon assimilation over time (Fig. S8B), suggesting increased escape or tolerance. Escape traits within *Mimulus guttatus* have been shown to evolve along with avoidance when growing seasons are shortened (Kooyers et al. 2015); this possibility should be further studied in *M. cardinalis* to further clarify these relationships. Although photosynthetic traits could be less responsive to intermittent perturbations than phenological and morphological traits, our study hints at the possibility of rapid evolution of photosynthetic traits over a short period of time and across populations.

### PLASTICTY AS A DROUGHT ADAPTATION

Plasticity in response to drought has been previously documented in resurrection studies (Franks 2011), yet it is less clear how plasticity can vary across the range of a species or how often plasticity is the target of selection. In our study, we find variability in plasticity across traits and regions, suggesting that plasticity could be important in certain contexts, but does not uniformly serve as an adaptation to drought. For example, we found substantial plasticity on the date of flowering that was maintained across the drought years in all regions (Fig. 2A‐C), but less plasticity in SLA (Fig. 2D‐F). Overall, the ability for plants in Mediterranean climates to react plastically to drought conditions could be an important context‐dependent strategy for survival within drought‐prone climates. Incorporating plasticity across environmental clines and particularly at range limits might be critical to more accurate species distribution modeling and forecasts for range shifts (Matesanz et al. 2010b). Alternatively, plasticity could be a non‐adaptive consequence of low water or resource availability.

### EVOLUTION OF PLASTICITY

There are two possibilities for why plasticity may have evolved throughout the drought. Trait plasticity can buffer environmental variability and thus serve as an adaptation to changing environmental conditions (Chevin et al. 2010; Nicotra et al. 2010; Anderson et al. 2012; Richter et al. 2012). In the context of a perennial species inhabiting a seasonally and topographically complex environment, this buffering could be important due to variability in water availability across a season, and between different years. Evolution of plasticity may also arise due to correlated selection since an individual trait that varies expression across two environments can be thought of as two different but correlated traits (Falconer 1996). Under this viewpoint, the evolution of trait expression in a wet environment may also occur not just because plasticity itself is favored, but also because of correlated evolution, where the dry environment produced plasticity as a byproduct.

It is important to characterize when and where plasticity in response to climate change occurs and how frequently plasticity itself evolves as an adaptation to novel stress conditions (Matesanz et al. 2010a). We found no evidence in support of evolution of increased plasticity in any region, and in fact evidence of canalization (loss of plasticity) in SLA in the south (Fig. 2F). Interestingly, this plasticity was lost over time, leading to a convergence of SLA values between wet and dry treatments at the height of the record‐setting drought (Fig. 2C). This could have occurred simply because of correlated evolution and strong selection for decreased SLA in the drought treatment. However, this result also matches previous predictions that evolutionary loss of plasticity could be adaptive (Matesanz et al. 2010b). Regardless of the exact mechanism by which loss of plasticity evolved any benefit at the height of the drought could come at the cost of less flexibility during less extreme conditions such as subsequent wet periods. This is especially true if genetic variation is lost, which is likely given lower population sizes in southern *M. cardinalis* at the height of the drought (Sheth & Angert 2018).

### REGIONAL DIFFERENCES

Characterizing local adaptation and clines in adaptive traits across environmental gradients and through the ranges of species has long been of interest to evolutionary biologists (Clausen et al. 1940; Dobzhansky 1950; Mayr 1956; MacArthur 1972). In recent decades there has been an increased interest in understanding how specific traits such as phenology (Olsson & Ågren 2002; Stinchcombe et al. 2004; Vitasse et al. 2009), photosynthesis (Hopkins et al. 2008; Muir & Angert 2017), anti‐herbivory defenses (Woods et al. 2012; Kooyers et al. 2017; Moreira et al. 2017; Moreira et al. 2018), temperature tolerance (Daday 1954; Hoffmann et al. 2002; Montesinos‐Navarro et al. 2011; Agren & Schemske 2012), and adaptations to drought (Paccard et al. 2014; Kooyers et al. 2015; Dickman et al. 2019) vary across space and their relationship with underlying environmental gradients. A critical question is how these clines in adaptive traits will be altered with the increasing intensity of climate change.

Resurrection experiments carried out across temperature and aridity gradients can establish latitudinal gradients in these traits prior to and after extreme climatic events. In our study, we observed considerably earlier flowering time in the southern region compared to the central and northern range, and a larger capacity for higher SLA in southern families grown under a dry treatment (Fig. 2). Across the length of our study, these clines disappear entirely for SLA and show a trend towards becoming weaker in the case of date of flowering. The impacts of climate change on clines in adaptive traits are understudied, yet might be vital in understanding how climate change is altering previous biogeographic patterns in adaptation and how this might impact the testing of macroecological hypotheses (Louthan et al. 2015; Anstett et al. 2016). Further characterization of change in adaptive clines may be facilitated by increasing availability of range‐wide seed collections, such as those from project baseline (Etterson *et al*. 2016).

In our study, the southern region diverged the most in terms of baseline trait values and their shift from pre‐ to peak‐drought. For four of the five measured traits, slopes of change over time were numerically greatest in the southern region (Fig. 3). This suggests stronger selection in southern regions and/or greater genetic variation in these traits. Moisture deficits in southern regions showed greater anomalies from average conditions for 30 years earlier (Fig. 1D), and PDSI was at record lows (Fig. S4A) suggesting that stronger selection could have played an important role. Consistent with greater genetic variation, southern populations showed the strongest response to artificial selection on phenology (Sheth & Angert 2016). Southern populations of *Mimulus cardinalis* could contain greater genetic variation due to a greater age of these populations (Hampe & Petit 2005) or perhaps because southern locations have microclimatic variation that provides a starker environmental contrast when compared to similar spatial scales in central and northern regions (personal observation). Additionally, a prior history of stress may facilitate rapid evolution and evolutionary rescue (Gonzalez & Bell 2013).

Central and northern populations also differed in their patterns, with northern populations showing less evidence of adaptive evolution when compared to the other regions (Fig. 2). It is possible that selection was not as intense in the north. Northern populations could have been limited by multiple genetic bottlenecks during past range expansion and simply had reduced genetic variability (Excoffier et al. 2009; Sheth & Angert 2016). In contrast, central locations may have had access to more genetic variation and greater linkages between populations, leading to more genetic variation that could allow for increased drought adaptations. *Mimulus cardinalis* has been shown to be both limited by climate and by dispersal at its northern range‐edge (Angert et al. 2018), making these hypotheses not mutually exclusive.

### CAVEATS

This study captures regional patterns in rapid evolution to drought across eleven populations, allowing for considerable spatial and temporal scope. This comes at the cost of having sampled 10 or fewer individuals from each site/year, which risks under sampling important genetic variation at any one site at a given point in time. This approach contrasts with other studies that are able to better sample a smaller number of populations (Franks et al. 2007; Hamann et al. 2018; Dickman et al. 2019). However, population sizes within *M. cardinalis* can often be small, increasing the likelihood that we are sampling enough of the population to encompass its genetic variation. As well, since *M. cardinalis* seeds are water dispersed, sampling at the most downstream point of each site bolsters our ability to sample representative site‐level genetic variation. Ultimately, the biggest strength in understanding regional patterns is our spatiotemporal spread, since when the data are analyzed together, we are able to mitigate uncertainty at any one site/year sampling point with many years of data and multiple sites sampled per region.

An additional point of interest is within‐plant variation of leaf traits. SLA, carbon assimilation, and stomatal conductance may be plastic throughout the plant. Leaves produced across the plant may vary in these traits depending on ontogeny and the level of drought exposure during development. Although this study does not directly consider this within‐plant source of variation, our replication within each family across different populations, and our consistent sampling of leaves within the central portions of the plant ensure our results are robust to this within plant variation. However, directly quantifying this variation may lead to more nuanced interpretations of results and further insights that are worth exploring.

### POSSIBILITY OF EVOLUTIONARY RESCUE

Climate change is a world‐wide threat to the maintenance of biodiversity. This threat is particularly acute for smaller populations in sensitive habitats, such as riparian corridors (Leung & Wigmosta 1999; Trivedi et al. 2008; Capon et al. 2013). Rapid evolution in response to extreme stresses such as drought may allow for populations to become adapted to warmer, drier, and more variable conditions (Franks et al. 2007; Dickman et al. 2019). However, rapid adaptation is only the first step in achieving evolutionary rescue. During evolutionary rescue, populations decline in size and then undergo recovery as individuals with adaptive alleles increase in frequency and allow population growth rate to increase (Gomulkiewicz & Holt 1995). During this period of decline and recovery, small populations are at increased risk of stochastic extinction (Gomulkiewicz & Houle 2009; Bell & Gonzalez 2011; Bell 2013). By the time drought was having a severe impact in 2014, populations of *M. cardinalis* were under decline despite demographic compensation, with southern range‐edge populations being most affected (Sheth & Angert 2018). It is unclear what the fate of these populations will ultimately be. Additional investigation into the impacts of the observed rapid evolution on population demography and trajectories would help establish if and when evolutionary rescue has occurred.

## AUTHOR CONTRIBUTIONS

D.A. and A.A. designed the experiment. D.A. and H.B. carried out data collection. D.A. and A.A. carried out the analysis with contributions from H.B. D.A. wrote the paper with edits from H.B. and A.A.

## CONFLICT OF INTEREST

The authors have declared no conflict of interest.

## DATA ARCHIVING

The data and R code for this project are available on our GitHub repository https://github.com/anstettd/Anstett_etal.2021. The data is also available on Dryad: https://doi.org/10.5061/dryad.jq2bvq881.

## Supporting information


**Figure S1**. Mean annual temperature and precipitation across the range of *M. cardinalis* during extreme drought and historical conditions
**Figure S2**. Percent germination per sampled accession across the 11 sites included in this study
**Figure S3**. Climate moisture deficit anomaly (CDMA) for each year/site combination across all 12 sites
**Figure S4**. Palmer drought severity index (PDSI) for across four drainages in California and Southwest Oregon
**Figure S5**. Mean annual temperature (MAT) for each year/site combination across all 11 target sites since 1980
**Figure S6**. Climate moisture deficit (CDM) for each year/site combination across all 11 target sites since 1980
**Figure S7**. Mean annual precipitation for each year/site combination across all 11 target sites since 1980
**Figure S8**. Evolution of water content, carbon assimilation, and stomatal conductance from the least to most drought‐impacted year
**Table S1**. The coordinates, elevation and region of studied sites.
**Table S2**. Per family germination percentages across all sites/year combinations
**Table S3**. Log likelihood assessment of Region*Year*Treatment + Random Effects models predicting SLA, date of flowering, water content, carbon assimilation, and photosynthetic stomatal conductance across the Californian megadrought
