# Supplementary material for: Regional differences in rapid evolution during severe drought
Source: Evol Lett. 2021 Feb 23;5(2):130–42. doi: 10.1002/evl3.218 (PMC8045920; doi:10.1002/evl3.218)
Supplement: Supplementary file 1 — Figure S1. Mean annual temperature and precipitation across the range of M. cardinalis during extreme drought and historical conditions Figure S2. Percent germination per sampled accession across the 11 sites included in this study Figure S3. Climate moisture deficit anomaly (CDMA) for each year/site combination across all 12 sites Figure S4. Palmer drought severity index (PDSI) for across four drainages in California and Southwest Oregon Figure S5. Mean annual temperature (MAT) for each year/site combination across all 11 target sites since 1980 Figure S6. Climate moisture deficit (CDM) for each year/site combination across all 11 target sites since 1980 Figure S7. Mean annual precipitation for each year/site combination across all 11 target sites since 1980 Figure S8. Evolution of water content, carbon assimilation, and stomatal conductance from the least to most drought‐impacted year Table S1. The coordinates, elevation and region of studied sites. Table S2. Per family germination percentages across all sites/year combinations Table S3. Log likelihood assessment of Region*Year*Treatment + Random Effects models predicting SLA, date of flowering, water content, carbon assimilation, and photosynthetic stomatal conductance across the Californian megadrought [file EVL3-5-130-s001.docx]

**Regional differences in rapid evolution during severe drought**

Daniel N. Anstett^1^, Haley A. Branch^1^, & Amy L. Angert^1,2^

**Figure S1**

Mean annual temperature and precipitation across the range of *M. cardinalis* during extreme drought and historical conditions. Sites are arranged by latitude and color coded by region (blue = North, orange = Centre, red = South). (A) The median and distribution of yearly mean annual temperature (MAT; °C) for each year between 1979 and 2009 for each site. Boxes represent the interquartile range, while the black line is the median. Dots are years further than 1.5 times the interquartile range. (B) Coefficient of variation for mean annual temperature experienced between 1979 and 2009 calculated in Kelvin. (C) Mean annual temperature anomaly (MATA; °C) during the studied drought cycle. The black line delineates no anomaly. Each point represents one year. The single digit numbers represent the last digit of the year (e.g. 0 = 2010, 4=2014). (D) The median and distribution of yearly mean annual precipitation (MAP; mm) for each year between 1979 and 2009 for each site. Boxes represent the interquartile range, while the black line is the median. Dots are years further than 1.5 times the interquartile range. (E) Coefficient of variation for mean annual precipitation experienced between 1979 and 2009. (F) Mean annual precipitation anomaly (MAPA, log mm) during the studied drought cycle. The black line delineates no anomaly. Each point represents one year. The single digit numbers represent the last digit of the year (e.g. 0 = 2010, 4=2014).

**Figure S2**

Percent germination per sampled accession across the 11 sites included in this study. Sites were sampled between 2010 to 2016, with most site/year combinations sampled.

**Figure S3**

Climate moisture deficit anomaly (CDMA) for each year/site combination across all 12 sites. Sites/year combinations were included in the analyses with the goal of testing adaptations from the lowest to highest CMDA. Site/year combinations included in the current study are shown as black circles, those removed from the study are shown in red circles, and those for which we never had seed material are in blue circles. Site 12 was removed from the study entirely since almost all sampled years had approximately the same CMDA and the peak CMDA year was not sampled.

**Figure S4**

Palmer drought severity index (PDSI) for across four drainages in California and Southwest Oregon. (A) South Coast includes sites 1 and 2; (B) San Joaquin includes sites 3-5; (C) Sacramento includes sites 6 and 7; (D) North Coast includes Sites 8 and 9; and (E) Southwest Oregon includes sites 10 and 11.

**Figure S5**

Mean annual temperature (MAT) for each year/site combination across all 11 target sites since 1980.

**Figure S6**

Climate moisture deficit (CDM) for each year/site combination across all 11 target sites since 1980.

**Figure S7**

Mean annual precipitation for each year/site combination across all 11 target sites since 1980. Site 8 contains a measurement anomaly with very lower precipitation likely due to poor precision of interpolation methods.

**Figure S8**

Evolution of water content, carbon assimilation, and stomatal conductance from the least to most drought-impacted year. There is relatively weak evidence of differences across regions and drought treatment levels. Each point represents residuals from a mixed model Region*Year*Treatment model with Site, Family and Block as random effects. The lines are linear models run on the residuals with 95% confidence intervals given for both well-watered and drought treatments.

Table S1 The coordinates, elevation and region of studied sites.

| Site | ID | Site Name | Region | Lat (°N) | Long (°W) | Elevation (m) |
| --- | --- | --- | --- | --- | --- | --- |
| 1 | S02 | Sweetwater River | South | 32.89928 | -116.5849 | 1168 |
| 2 | S07 | West Fork Mojave River | South | 34.28425 | -117.37539 | 1092 |
| 3 | S10 | North Fork Middle Fork Tule | Centre | 36.20081 | -118.65092 | 1288 |
| 4 | S08 | Redwood Creek | Centre | 36.69096 | -118.90961 | 1682 |
| 5 | S32 | Wawona | Centre | 37.539 | -119.654 | 1214 |
| 6 | S29 | Oregon Creek | Centre | 39.39442 | -121.08302 | 440 |
| 7 | S18 | Little Jameson Creek | Centre | 39.74298 | -120.70401 | 1593 |
| 8 | S17 | Deep Creek | North | 41.66546 | -123.11341 | 680 |
| 9 | S16 | O'Neil Creek | North | 41.80979 | -123.11887 | 1092 |
| 10 | S36 | Deer Creek | North | 42.27411 | -123.63617 | 393 |
| 11 | S15 | Rock Creek | North | 43.37876 | -122.95207 | 1168 |

Table S2 Per family germination percentages across all sites/year combinations. Number of fruits without germination, with germination, and total number of fruits are also given.

| **Site** | **Year** | **No Germination** | **Germination** | **Total** | **Percent Germination** |
| --- | --- | --- | --- | --- | --- |
| 1 | 2010 | 2 | 8 | 10 | 80 |
| 1 | 2011 | 2 | 7 | 9 | 77.8 |
| 1 | 2012 | 0 | 7 | 7 | 100 |
| 1 | 2013 | 1 | 6 | 7 | 85.7 |
| 1 | 2014 | 1 | 10 | 11 | 90.9 |
| 2 | 2010 | 6 | 4 | 10 | 40 |
| 2 | 2011 | 4 | 7 | 11 | 63.6 |
| 2 | 2014 | 4 | 14 | 18 | 77.8 |
| 2 | 2015 | 0 | 7 | 7 | 100 |
| 2 | 2016 | 4 | 3 | 7 | 42.9 |
| 3 | 2010 | 7 | 2 | 9 | 22.2 |
| 3 | 2011 | 3 | 2 | 5 | 40 |
| 3 | 2012 | 0 | 3 | 3 | 100 |
| 3 | 2013 | 0 | 5 | 5 | 100 |
| 3 | 2014 | 2 | 9 | 11 | 81.8 |
| 3 | 2015 | 2 | 9 | 11 | 81.8 |
| 3 | 2016 | 7 | 4 | 11 | 36.4 |
| 4 | 2011 | 2 | 3 | 5 | 60 |
| 4 | 2012 | 3 | 10 | 13 | 76.9 |
| 4 | 2013 | 1 | 6 | 7 | 85.7 |
| 4 | 2014 | 4 | 1 | 5 | 20 |
| 5 | 2010 | 5 | 11 | 16 | 68.8 |
| 5 | 2012 | 0 | 10 | 10 | 100 |
| 5 | 2014 | 2 | 6 | 8 | 75 |
| 5 | 2015 | 3 | 6 | 9 | 66.7 |
| 5 | 2016 | 1 | 6 | 7 | 85.7 |
| 6 | 2010 | 1 | 8 | 9 | 88.9 |
| 6 | 2013 | 2 | 2 | 4 | 50 |
| 6 | 2014 | 1 | 8 | 9 | 88.9 |
| 6 | 2015 | 0 | 3 | 3 | 100 |
| 6 | 2016 | 3 | 1 | 4 | 25 |
| 7 | 2010 | 4 | 8 | 12 | 66.7 |
| 7 | 2012 | 0 | 8 | 8 | 100 |
| 7 | 2013 | 1 | 10 | 11 | 90.9 |
| 7 | 2014 | 0 | 9 | 9 | 100 |
| 7 | 2016 | 1 | 9 | 10 | 90 |
| 8 | 2011 | 5 | 4 | 9 | 44.4 |
| 8 | 2014 | 0 | 1 | 1 | 100 |
| 8 | 2015 | 5 | 5 | 10 | 50 |
| 8 | 2016 | 1 | 9 | 10 | 90 |
| 9 | 2010 | 6 | 8 | 14 | 57.1 |
| 9 | 2012 | 1 | 4 | 5 | 80 |
| 9 | 2013 | 0 | 4 | 4 | 100 |
| 9 | 2014 | 5 | 5 | 10 | 50 |
| 9 | 2015 | 6 | 7 | 13 | 53.8 |
| 9 | 2016 | 0 | 10 | 10 | 100 |
| 10 | 2011 | 0 | 10 | 10 | 100 |
| 10 | 2012 | 0 | 11 | 11 | 100 |
| 10 | 2014 | 2 | 8 | 10 | 80 |
| 10 | 2015 | 2 | 9 | 11 | 81.8 |
| 10 | 2016 | 1 | 9 | 10 | 90 |
| 11 | 2010 | 3 | 9 | 12 | 75 |
| 11 | 2012 | 1 | 2 | 3 | 66.7 |
| 11 | 2014 | 5 | 5 | 10 | 50 |
| 11 | 2015 | 1 | 9 | 10 | 90 |
| 11 | 2016 | 0 | 21 | 21 | 100 |

Table S3 Log likelihood assessment of Region*Year*Treatment + Random Effects models predicting SLA, date of flowering, water content, carbon assimilation, and photosynthetic stomatal conductance across the Californian megadrought. Significant p-values (P<0.05) and highest significant log likelihood are given in bold. Marginally significant values (0.05<P<0.10) are given in italics. Selected model is given in red.

| Trait | Fixed Effects | Log Likelihood | P-value |
| --- | --- | --- | --- |
| SLA | **Region*Year*Treatment** | **-8849.4** | **0.003** |
|  | Region * Treatment + Treatment * Year + Region * Year | -8855.2 |  |
| Date of Flowering | Region*Year*Treatment | -6920.9 | 0.22 |
|  | Region * Treatment + Treatment * Year + Region * Year | -6922.4 |  |
|  |  |  |  |
| 1 | **Region * Treatment + Treatment * Year + Region * Year** | **-6922.4** | **0.11** |
|  | Treatment * Year + Region * Year | -6926.9 |  |
|  |  |  |  |
| 2 | Region * Treatment + Treatment * Year + Region * Year | -6922.4 | 0.53 |
|  | Region * Treatment + Region * Year | -6922.2 |  |
|  |  |  |  |
| A | **Region * Treatment + Region * Year** | **-6922.2** | **0.014** |
|  | Region * Year + Treatment | -6926.5 |  |
|  |  |  |  |
| B | Region * Treatment + Region * Year | -6922.2 | 0.772 |
|  | Region * Treatment + Year | -6922.5 |  |
|  |  |  |  |
|  | **Region * Treatment + Year** | **-6922.5** | **0.014** |
|  | Region + Treatment + Year | -6926.7 |  |
|  |  |  |  |
| 3 | Region * Treatment + Treatment * Year + Region * Year | -6922.4 | 0.78 |
|  | Region * Treatment + Treatment * Year | -6922.7 |  |
|  |  |  |  |
| A | **Region * Treatment + Treatment * Year** | **-6922.7** | **0.01** |
|  | Treatment * Year + Region | -6927.1 |  |
|  |  |  |  |
| B | Region * Treatment + Treatment * Year | -6922.7 | 0.54 |
|  | **Region * Treatment + Year** | -6922.5 |  |
| Water Content | Region*Year*Treatment | 2467.4 | **<0.001** |
|  | **Region * Treatment + Treatment * Year + Region * Year** | **2476.4** |  |
|  |  |  |  |
|  | Region * Treatment + Treatment * Year + Region * Year | 2476.4 | **0.001** |
|  | **Region * Treatment + Region * Year** | **2481.8** |  |
|  |  |  |  |
|  | Region * Treatment + Region * Year | 2481.8 | **<0.001** |
|  | **Region * Year + Treatment** | **2489.3** |  |
|  |  |  |  |
|  | Region * Year + Treatment | 2489.3 | **<0.001** |
|  | **Region + Year + Treatment** | **2496.9** |  |
|  |  |  |  |
|  | Region + Year + Treatment | 2496.9 | 0.152 |
|  | Year + Treatment | 2495.1 |  |
|  |  |  |  |
|  | **Year + Treatment** | **2495.1** | **0.001** |
|  | Year | 2489.9 |  |
|  |  |  |  |
|  | Year + Treatment | 2495.1 | **<0.001** |
|  | **Treatment** | **2501.2** |  |
|  |  |  |  |
|  | **Treatment** | **2501.2** | **0.001** |
|  | Random Effects Only | 2496 |  |
| Assimilation | Region*Year*Treatment | -2370.9 | 0.89 |
|  | Region * Treatment + Treatment * Year + Region * Year | -2370.8 |  |
|  |  |  |  |
| 1 | ***Region * Treatment + Treatment * Year + Region * Year*** | **-2370.8** | *0.063* |
|  | Treatment * Year + Region * Year | -2373.6 |  |
|  |  |  |  |
| 2 | Region * Treatment + Treatment * Year + Region * Year | -2370.8 | 0.14 |
|  | Region * Treatment + Region * Year | -2369.7 |  |
|  |  |  |  |
| A | **Region * Treatment + Region * Year** | **-2369.7** | *0.06* |
|  | Region * Year + Treatment | -2372.5 |  |
|  |  |  |  |
| B | Region * Treatment + Region * Year | -2369.7 | 0.47 |
|  | Region * Treatment + Year | -2369 |  |
|  |  |  |  |
|  | **Region * Treatment + Year** | **-2369** | *0.06* |
|  | Region + Treatment + Year | -2371.8 |  |
|  |  |  |  |
| 3 | Region * Treatment + Treatment * Year + Region * Year | -2370.8 | 0.47 |
|  | Region * Treatment + Treatment * Year | -2370.1 |  |
|  |  |  |  |
| A | **Region * Treatment + Treatment * Year** | **-2370.1** | *0.06* |
|  | Treatment * Year + Region | -2372.8 |  |
|  |  |  |  |
| B | Region * Treatment + Treatment * Year | -2370.1 | 0.14 |
|  | Region * Treatment + Year | -2369 |  |
| Stomatal Conductance | Region*Year*Treatment | 185.51 | **0.003** |
|  | **Region * Treatment + Treatment * Year + Region * Year** | **191.46** |  |
|  |  |  |  |
|  | Region * Treatment + Treatment * Year + Region * Year | 191.46 | **0.006** |
|  | **Region * Treatment + Region * Year** | ***195.18*** |  |
|  |  |  |  |
|  | Region * Treatment + Region * Year | 195.18 | **0.038** |
|  | **Region * Year + Treatment** | **198.44** |  |
|  |  |  |  |
|  | Region * Year + Treatment | 198.44 | **0.002** |
|  | **Region + Year + Treatment** | **204.52** |  |
|  |  |  |  |
|  | Region + Year + Treatment | 204.52 | **0.01** |
|  | Year + Treatment | **209.15** |  |
|  |  |  |  |
|  | *Year + Treatment* | *209.15* | *0.057* |
|  | Year | 207.34 |  |
|  |  |  |  |
|  | Year + Treatment | 209.15 | **0.003** |
|  | **Treatment** | **213.45** |  |
|  |  |  |  |
|  | **Treatment** | **213.45** | *0.055* |
|  | Random Effects Only | 211.6 |  |
